# Using protein microarray technology to screen anti-ERCC1 monoclonal antibodies for specificity and applications in pathology

**DOI:** 10.1186/1472-6750-12-88

**Published:** 2012-11-21

**Authors:** Donghui Ma, Dror Baruch, Youmin Shu, Kehu Yuan, Zairen Sun, Kaiyan Ma, Toan Hoang, Wei Fu, Li Min, Zhu-Sheng Lan, Fangxun Wang, Lori Mull, Wei-Wu He

**Affiliations:** 1 OriGene Technologies Inc, 9620 Medical Center Drive, Rockville, Maryland, 20850, USA

## Abstract

**Background:**

An antibody with cross-reactivity can create unexpected side effects or false diagnostic reports if used for clinical purposes. ERCC1 is being explored as a predictive diagnostic biomarker for cisplatin-based chemotherapy. High ERCC1 expression is linked to drug resistance on cisplatin-based chemotherapy. 8F1 is one of the most commonly used monoclonal antibodies for evaluating ERCC1 expression levels in lung cancer patient tissues, but it has been noted that this antibody cross-reacts with an unknown protein.

**Results:**

By using a high density protein microarray chip technology, we discovered that 8F1 not only reacts with its authentic target, ERCC1, but also cross-reacts with an unrelated nuclear membrane protein, PCYT1A. The cross-reactivity is due to a common epitope presented on these two unrelated proteins. Similar to the subcellular localization of ERCC1, IHC tests demonstrated that PCYT1A is localized mainly on nuclear membrane. In this study, we also discovered that the PCYT1A gene expression level is significantly higher than the ERCC1 gene expression level in a certain population of lung cancer patient tissue samples. To develop the best monoclonal antibody for ERCC1 IHC analysis, 18 monoclonal antibodies were generated and 6 of them were screened against our protein microarray chip. Two clones showed high mono-specificity on the protein microarray chip test and both worked for the IHC application.

**Conclusion:**

In summary, the 8F1 clone is not suitable for ERCC1 IHC assay due to its cross-reactivity with PCYT1A protein. Two newly generated monoclonal antibodies, 4F9 and 2E12, demonstrated ultra-specificity against ERCC1 protein and superior performance for IHC analyses.

## Background

Non-small cell lung cancer (NSCLC) is the most common form of lung cancer. It accounts for more than 80% of all lung cancer related deaths [1]. After surgical removal of NSCLC, cisplatin-based chemotherapy is the first line of treatment [2]. Cisplatin and its derivatives are mutagenic chemicals that can induce DNA interstrand or intrastrand cross links through forming DNA adducts. Tumor cells damaged by these drugs will trigger cell death [3]. However, cisplatin-based chemotherapy also causes highly toxic side effects [4-7]. Recent statistical analysis of clinical data shows that cisplatin-based chemotherapy can only add an approximately 4–15% survival advantage at five years with a subgroup of NSCLC patients exhibiting no beneficial effects of drug treatment or even worse outcomes after treatment [8,9]. Thus, it is imperative to use prognostic or predictive biomarkers to identify patients who should receive cisplatin-based chemotherapy [10].

Cisplatin or other DNA crosslinkers could cause DNA damages on either one or two strands of DNA duplex. In cells, multiple DNA repair pathways are involved in repairing these damages, including nucleotide excision repair and interstrand crosslink repair pathway [11]. Therefore, strong endogenous DNA damage repairing capabilities in tumor cells will compromise the therapeutic drug effects of cisplatin [12]. This could be the major biochemical mechanism that explains different patient responses towards cisplatin-based treatments [13]. The ERCC1-XPF heterodimeric endonuclease protein complex involves in both nucleotide excision repair and interstrand crosslink repair pathway. XPF acts as the catalytic subunit and ERCC1 binds DNA [14]. Quite a number of clinical studies have already shown a good correlation between ERCC1 mRNA level and cisplatin drug resistance [15-17]. This suggests that examining ERCC1 expression levels might be a good method to identify patients who are likely to benefit from cisplatin-based chemotherapy.

To evaluate ERCC1 protein levels in formalin-fixed paraffin-embedded tissue samples, a high quality monoclonal antibody validated for immunohistochemistry is needed. Currently there are several anti-ERCC1 IHC antibodies available on the market [18,19]. However, the published data for them is highly controversial and none of them is widely accepted as specific for application in IHC. More than 40 published clinical studies were conducted using the 8F1 clone making it the most commonly used anti-ERCC1 diagnostic monoclonal antibody currently on the market [20-24]. Recently, its target specificity has been questioned [19,25]. By using an ERCC1-XPF deficient cell line, Bhagwat et al. discovered that the 8F1 antibody binds to an unknown cross-reactive protein that migrates at a molecular weight similar to ERCC1 on SDS-PAGE gels but the molecular identity for this cross-reactive target was unknown [19]. In this paper, we describe the development and application of a high density protein microarray chip. With this technology, we were not only able to identify the molecular identity of 8F1 cross-reactive protein, but also able to use it as a tool to develop two highly specific monoclonal antibodies for ERCC1 IHC assay.

## Methods

### The high-throughput production of overexpression lysates from HEK293T cells transiently transfected with ORF cDNA expression clones

HEK293T cells from four fully confluent T150 tissue culture flasks were trypsinized and inoculated into 96 10 cm-tissue culture plates. After culturing for 24 hrs, cells were transiently transfected with 5ug of TrueORF cDNA plasmid per plate. After transfection, the cells were cultured at 37°C for another 48 hrs. Before cell lysate collection, transfected cells were washed once with 1xDPBS. Next, 800ul of freshly-prepared native RIPA lysis buffer (25 mM Tris-HCl pH7.6, 150 mM NaCl, 1% NP-40, 1 mM EDTA, 1xProtein inhibitor cocktail mix, 1 mM PMSF and 1 mM Na3VO4) is added directly to the cells and the plates were incubated on ice for 5 minutes to lyse the cells completely. The 96 crude cell lysates were transferred to a 96-deep well plate with 2 ml capacity (Axygen) and spun at 4000 x rpm to remove insoluble cellular debris. The clarified supernatants were transferred to a 96-well v-shape plate (BD) and stored at -80C for future protein microarray printing. The recombinant protein expression for every overexpression lysate used for microarray printing was validated by Western-blot analysis with the anti-DDK antibody (OriGene, TA50011-100).

### Protein microarray printing

All overexpression lysates used for array printing were obtained from OriGene Technologies Inc. The detailed protocol for overexpression lysate production is described as above. Protein microarray printing was performed with QArray2 array printer (Genetix) with 48 5 um XSMP2 split pins. Grace Biolab Ovid 20X60 mm nitrocellulose slides were used for printing. The array contains a total of 22,176 spots with 10,464 lysates in duplicates (20,928 spots) and 1248 control spots. The whole slide is divided into 48 sub-arrays (4X12). For each subarray, 462 samples were spotted in 22X21 format. As an orientation marker control, each subarray contains an autofluorescent BSA-Cy5 and Cy3 mixture spot and a mixed IgG spot (mixture of human, mouse and rabbit IgGs). There are also additional sub-array controls including a buffer only negative control, a 1 mg/ml BSA control and a 1 mg/ml HEK293T lysate control. In addition to above subarray controls, we also added specific global positive and quantification controls. These controls include serially-diluted IgG mixture controls placed in sub-arrays C1, C5 and C9. As global negative control, serially diluted HEK293T lysates were spotted in subarrays A1, A5 and A9. Since every cDNA clone used for overexpression lysate production contains a DDK epitope tag, the recombinant protein expression level can be detected by using the 4C5 monoclonal anti-DDK antibody (TA50011-100, OriGene Technologies Inc.). Because the protein expression level for different genes varies greatly, two highly purified DDK fusion proteins were used as reference standards for the purpose of quantification. These two standards are GST and beta-actin. They are located either in subarray D1, D5 and D9 for GST or subarray B1, B5 and B9 for beta-actin.

### Protein microarray immuno-hybridization assay

Before probing, the array chips were hydrated for at least 30 min in 10 ml distilled water followed by a 5 min equilibration with 5 ml wash buffer (20 mMTris, 150 mMNaCl, 0.05% Tween 20. pH 8.0).

Primary antibody was diluted to 5–10 μg/ml in 1 ml blocking buffer (StartingBlock T20 (TBS), Thermo) and added to the array in a ProPlate chamber (Grace-bio). The arrays were then incubated overnight at 5C with rocking. After overnight antibody incubation, arrays were washed briefly with 2 ml of wash buffer, transferred to Perfect Western 6-sectional short (B130) slide tray and then washed three times with 5 ml of wash buffer for 5 minutes each time.

DyLight 649-conjugated secondary antibody (Jackson Labs) was diluted 1:500 in wash buffer, added to the array slide, and then incubated for another 30 min at RT with rocking. After incubation, the stained arrays were washed extensively with distilled water and air-dried. The arrays were scanned with a Genepix 4100A scanner and data were analyzed using the appropriate gal file. The antibodies used for protein microarray chip analysis include rabbit polyclonal anti-ERCC1 (FL297) (Santa Cruz), anti-ERCC1 (8F1) (Abcam), and Anti-ERCC1 (4F9 and 2E12) (OriGene Technologies Inc.)

### Tissue microarray production and Immunohistochemistry

The tissue microarray chip was generated using Sakura’s Tissue-Tek Quick-Ray system. On one slide, 12 human normal and 12 human carcinoma tissue samples were spotted. For immunohistochemical staining of the tissue-microarray chip and paraffin-embedded tissue sections, antigen retrieval was carried out in 0.01 M Sodium Citrate buffer at pH 6 in a pressure cooker for 2 minutes. Samples were blocked with 5% non-fat milk plus 5% goat serum for 30 min and then incubated with primary antibody at a 1:150 dilution for 60 min at room temperature. The primary antibody signal was detected using polink-2 anti-rabbit or anti-mouse secondary antibody for 30 min and DAB substrate for 5 min at room temperature (GBI Labs). Hematoxylin was used for counterstaining. The antibodies used for IHC analysis include anti-PCYT1A (EPR3940) (Epitomics), anti-ERCC1 (8F1) (Abcam), Anti-ERCC1 (4F9 and 2E12) (OriGene Technologies Inc.).

### Quantitative real-time PCR

TissueScan 24 lung cancer panels (HLRT104, OriGene Technologies) were used to examine ERCC1 and PCYT1A mRNA expression profiles. The qPCR was performed on ABI 7900HT using the SYBR Green PCR Master Mix (Applied Biosystems). Primers used for ERCC1 mRNA detection were HP229725 with 5^′^-GCTGGCTAAGATGTGTATCCTGG-3^′^ (forward) and 5-ATCAGGAGGTCCGCTGGTTTCT-3 (reverse), for PCYT1A were HP208235 with 5^′^-GTTCCTTCCAAAGTGCAGCGCT-3^′^ (forward) and 5-AGGAGTTCCTCTGCTGGCTTCT-3, for β-actin were HP204660 with 5^′^-CAGCCATGTACGTTGCTATCCAGG-3^′^ (forward) and 5-AGGTCCAGACGCAGGATGGCATG-3 (OriGene Technologies Inc.). Relative quantification of ERCC1 and PCYT1A expression were measured by normalization against β-actin using the ΔCT method. The PCR efficiency values of E(ERCC1), E(PCYT1A) and E(β-actin)were determined using the copy number standards, HK202848, HK209617 and HK200550 respectively (OriGene Technologies Inc.).

### Immunoblotting and recombinant protein affinity purification

For immunoblotting, overexpression lysates (OriGene Technologies Inc.) or purified proteins were mixed with 4XSDS sample buffer (Invitrogen), boiled for 5 minutes before loading on SDS-PAGE for fractionation. After separation on SDS-PAGE gel, they were electro-blotted on a nitrocellulose membrane. Anti-DDK (OriGene Technologies Inc.), anti-ERCC1 (8F1) (Abcam) or anti-ERCC1 (4F9 or 2E12) (OriGene Technologies Inc.) was used for immunoblotting.

For affinity purification of recombinant protein, HEK293T cells were first transiently transfected with an OriGene TrueORF cDNA clone (OriGene Technologies Inc.). After 48 hrs, transfected cells were collected and lysed with RIPA buffer (25 mM Tris-HCl pH7.6, 150 mM NaCl, 1% NP-40, 1xProtein inhibitor cocktail mix, 1 mM PMSF, 1 mM EDTA and 1 mM Na3VO4) and the cellular debris were removed by high speed centrifugation. For affinity purification, anti-DDK agarose beads were first balanced with RIPA buffer, and then added to pre-cleared overexpression lysates for overnight incubation. After incubation, the beads were extensively washed and purified proteins were eluted from the beads using a 0.1 M Glycine-HCl (pH2.3) solution.

## Results

### Development of a high density overexpression lysate protein microarray chip for the evaluation of antibody specificity

**Figure 1:**
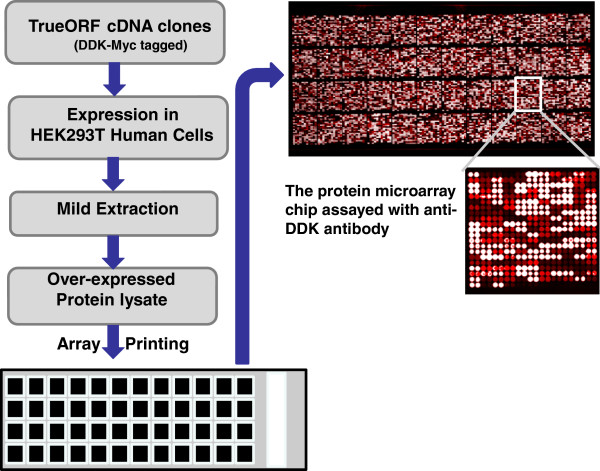
**The production scheme for the protein microarray chip.** All the overexpression lysates were produced using OriGene’s TrueORF cDNA clone collection. On a single nitrocellulose slide, over 22,000 protein samples were spotted. These include 10,464 unique gene overexpression lysates printed in duplicate and large selections of positive and negative controls. Since all the expression clones contain a universal Myc and DDK fusion tag, the target gene expression level can be examined by using an Anti-DDK antibody (1:500 dilution of OriGene anti-DDK (TA50011) followed by DyLight 649 conjugated goat anti-mouse IgG secondary antibody for detection.

**Figure 2:**
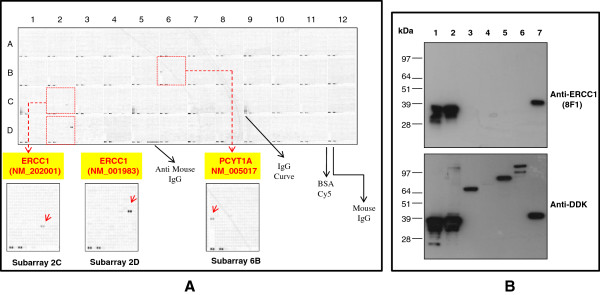
**The identification of the 8F1 cross-reactive protein with protein microarray chip and Western blot confirmation. A**. Protein microarray hybridization with 8F1. The OriGene overexpression protein microarray chip was immunostained with the most commonly used 8F1 monoclonal anti-ERCC1 antibody. The positive reactive proteins are highlighted with red arrows. These data show that 8F1 recognizes not only its specific target (two ERCC1 transcript variants), but also another unrelated nuclear membrane protein PCYT1A. A number of internal controls were also labeled on the panel. **B**. Western Blot analysis. Seven OriGene VERIFY™ overexpression lysate antigen standards (Lane 1 to 7) were fractionated on SDS-PAGE, and then immunoblotted with 8F1 (Upper panel). The recombinant protein expression levels within the lysates were analyzed with anti-DDK antibody (Lower panel). Lanes 1 to 7 are loaded with samples for ERCC1 (NM_202001), ERCC1 (NM_001983), ERCC2 (NM_000400), ERCC3 (NM_000122), ERCC4 (NM_005236), ERCC5 (NM_000123) and PCYT1A (NM_005017).

Figure 1 is the basic production scheme for this protein microarray chip. In summary, 10,464 unique overexpression lysates were spotted in duplicate on a single nitrocellulose coated glass slide. The detailed description of protein microarray production and calibration is provided in the materials and methods section. To ensure that the immunoassay experiments with these chips are reproducible and interpretable, multiple internal controls were spotted on the chip (Figure 2A).

### Using a high density protein microarray chip to evaluate the target specificity of anti-ERCC1 monoclonal antibodies

To evaluate the 8F1 target specificity, this antibody was incubated at 5ug/ml concentration with the protein microarray chip. As suspected from Bhagwat et al, 8F1 not only reacts with its authentic target ERCC1 protein, but also binds strongly with an un-related nuclear protein, PCYT1A (see subarray 6B in Figure 2A). PCYT1A is an important enzyme in regulating phosphatidylcholine biosynthesis and nuclear membrane expansion [26]. The functionality of this protein is actively regulated by a rapid translocation between a cytoplasmic soluble form that is inactive and a nuclear membrane-associated form that is activated [27]. No published data show any correlation between PCYT1A expression levels and patient response to cisplatin-based chemotherapy.

Rabbit polyclonal antibody FL297 is another commonly used anti-ERCC1 IHC antibody. Studies from a number of groups suggested that the target specificity for FL297 could be considerably better than 8F1 under their IHC experimental conditions [18,19,28,29]. To evaluate the specificity of this antibody, protein microarray chip hybridization and Western blot confirmation were performed. In comparison with the 8F1 monoclonal antibody, this rabbit antibody exhibits much broader cross-reactivity and higher background signals across the entire chip (see Additional file 1: Figure S1). On subarrays 2C and 2D, FL297 reacts with two ERCC1 recombinant protein isoforms encoded by two different transcript variants. However, it also shows strong cross-reactivity with two unrelated protein targets, PDK1 and FERMT3. This cross-reactivity was confirmed by WB analyses of PDK1 and FERMT3 overexpression lysates (Additional file 2: Figure S2). PDK1 is a central mediator of cellular signaling between PI-3 kinase and various intracellular serine/threonine kinases, including protein kinase B, p70 ribosomal S6 kinase, serum and glucocorticoid-inducible kinase, and protein kinase C. FERMT3 is a focal adhesion molecule implicated in integrin activation. So far there are no published results linking the protein or gene expression levels of either PDK1 or FERMT3 with the patient response to cisplatin-based chemotherapy. In terms of subcellular localization, neither of these two cross-reactive proteins is located in the nuclear region. These data are well correlated with previous observations that IHC immunostaining with FL297 shows some cytoplasmic staining [18].

### Immunoassay analysis to confirm the protein microarray hybridization data with cross-reactive targets

To further confirm the protein microarray data, Western blot analyses were performed with OriGene’s VERIFY™ overexpression lysates. As negative controls, we included 4 different ERCC family members on the same WB to confirm the antibody specificity. Figure 2B is the representative WB data with clone 8F1 (Figure 2B upper panel). From this data, it is clear that 8F1 not only recognizes ERCC1 protein, but also PCYT1A strongly. No cross-reactivity was observed with other ERCC family members. The WB membrane was also re-blotted with anti-DDK antibody to confirm the expression of the different recombinant proteins for each of the input lysates (Figure 2B lower panel).

**Figure 3:**
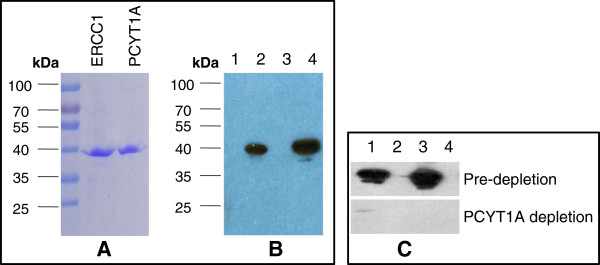
**8F1 immunoblot analyses with purified proteins and antigen absorption test. A**. Coomassie staining of purified ERCC1 and PCYT1A proteins. 1ug of affinity purified recombinant ERCC1 and PCYT1A proteins were fractionated on the SDS-PAGE gel and then commassie-stained. **B**. Immunoblot analysis with 8F1 anti-ERCC1 mAb.0.5ug of purified ERCC1 (Lane 2) and PCYT1A (Lane 4) were loaded on an SDS-PAGE gel and then immunobloted with 8F1 antibody. Empty vector transfected HEK293T cell lysates (Lanes 1 and 3) were used as a negative control. **C**. Antigen absorption test. Overexpression lysates for PCYT1A (Lane1), ERCC1 (Lane 3), and empty vector transfected control (Lanes 2 and 4) were fractionated on SDS-PAGE, and then immunoblotted with 8F1 (upper panel) or 8F1 pre-depleted with purified PCYT1A protein (lower panels).

One of the intrinsic questions with this WB data is that the SDS-PAGE gel migration location of ERCC1 and PCYT1A molecule is barely distinguishable, even though PCYT1A is 10 kDa larger than ERCC1. This observation is well correlated with the previous publication from Bhagwat NR, et al [19]. By using an ERCC1 depleted cell line, they detected an unidentified 8F1 cross-reactive nuclear protein with similar molecular weight. Although this data could be explained as the abnormal SDS-PAGE migration pattern of PCYT1A protein itself, it is also possible that the observed WB signal could be due to up-regulated endogenous ERCC1 protein upon transient overexpression of PCYT1A. To distinguish between these possibilities, we performed immunoassays with highly purified recombinant proteins instead of crude overexpression cell lysates. The SDS-PAGE gel electrophoresis data in Figure 3A shows that the highly purified ERCC1 and PCYT1A recombinant proteins exhibit very similar migration patterns. In order to compare the immunoreactive signal strength between 8F1 and these two highly purified recombinant proteins, equivalent amounts of protein were loaded on each lane for WB analysis. Interestingly, our data shows that WB band intensity is also nearly identical (Figure 3B). These data demonstrate that 8F1indeed binds PCYT1A protein with similar affinity to its authentic target, ERCC1 protein.

One possible explanation for this antibody cross-reactivity could be high sequence homology between its authentic target and the cross-reactive protein. To understand whether there is any amino acid sequence homology, we carried out an amino acid sequence alignment of ERCC1 and PCYT1A. No significant homology was detected (data not shown). However, it is possible that the epitope recognized by 8F1 is a discontinuous epitope and that a similar conformational epitope exists on PCYT1A because 8F1 was generated by using full length human recombinant ERCC1 protein as immunogen.

Monoclonal antibodies are produced by hybridoma cell lines, which are generated by the fusion between an antibody secreting B cell and a myeloma cell. Whether multiple B cells can fuse with one myeloma cell and produce different antibodies from a single hybridoma clone is still under-debate [30,31]. If this does occur, it raises the possibility that multiple antibodies can be secreted from one hybridoma. Alternatively, hybridoma clone impurity could also attribute to the contamination of 8F1 antibody preparation. To evaluate contamination as the source of cross-reactivity, a set of immunogen absorption experiments were performed. As shown in Figure 3C, the immunoreactivities toward both PCYT1A and ERCC1 were ablated effectively after the pre-absorption with purified PCYT1A protein. However, no signal depletion was observed under the control experimental condition that used purified BSA protein as the negative control (data not shown). These data clearly demonstrate that a single 8F1 IgG molecule exhibits bi-specific binding capacity towards both ERCC1 and PCYT1A protein.

### The immunohistochemical staining of NSCLC tissue sections with anti-ERCC1 and anti-PCYT1A monoclonal antibodies

ERCC1 protein is proposed as an important biomarker for clinicians to predict whether a certain patient population with non-small cell lung carcinoma (NSCLC) will respond to cisplatin chemotherapy [9]. For pathological evaluation via the application of IHC analysis, nuclear immunostaining intensity is the critical measurement.

The 8F1 cross-reactive protein, PCYT1A, is a nuclear membrane protein. However, previous immunocytochemistry studies indicated that the subcellular localization for PCYT1A is dynamically regulated under different physiological conditions and it can switch between an inactive soluble or cytoplasmic form to an active, membrane-bound species within the nucleus [27]. There are very few reports of immunohistochemistry analysis of PCYT1A protein in different tissues and those data are highly controversial [32].

**Figure 4:**
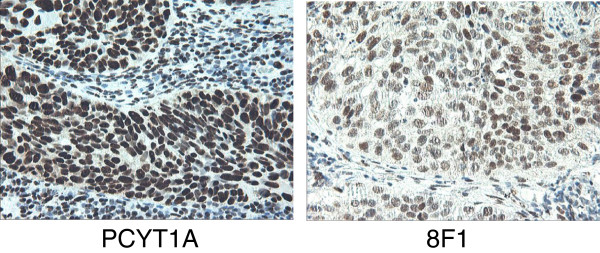
**The immunohistochemistry staining on NSCLC tissue sections with rabbit monoclonal anti-PCYT1A antibody or 8F1 mouse monoclonal anti-ERCC1 antibody.** Pathologist-validated NSCLC FFPE tissue blocks were cut into thin sections, and then further deparaffinized, re-hydrated before IHC testing. The primary antibodies (Left: rabbit anti-PCYT1A mAb, Right: 8F1 anti-ERCC1 mAb) used for these experiments were diluted at 1:150 dilution with blocking buffer.

To check the subcellular distribution pattern for PCYT1A protein in NSCLC tissue samples, IHC experiments were performed in parallel using either the rabbit monoclonal anti-PCYT1A or mouse 8F1 anti-ERCC1 antibody. Representative IHC images are in Figure 4. These data clearly show that PCYT1A IHC staining is localized primarily in the nucleus compartment and that the staining pattern is very similar to that seen with the anti-ERCC1 8F1 clone.

To further survey the PCYT1A protein tissue expression profile, IHC analyses were performed on a tissue microarray slide containing 12 normal human tissue samples and 12 corresponding human tumor tissue samples. The IHC data with positive staining are shown in Additional file 3: Figures S3A and Additional file 4: Figure S3B. In summary, PCYT1A is primarily localized within nucleus under our current experimental conditions.

### Comparison of ERCC1 and PCYT1A mRNA transcript levels by qPCR analysis on lung cancer patient tissue samples

Western blot analysis showed that there is no significant difference in terms of the binding efficiency for 8F1 to ERCC1 or to PCYT1A and our IHC data also demonstrated that ERCC1 and PCYT1A protein are colocalized within the nucleus compartment in NSCLC patient tissue samples. To facilitate our understanding of how much 8F1 nuclear immunostaining signal might be coming from 8F1-PCYT1A interaction, we wanted to compare the endogenous protein expression levels for PCYT1A and ERCC1 in lung cancer patients. However, the binding efficiency difference between each antibody-antigen pair would make it exceedingly difficult to estimate the endogenous protein expression level based on immunoassay data. Thus, we used a qPCR method to compare the endogenous expression at the mRNA level.

**Figure 5:**
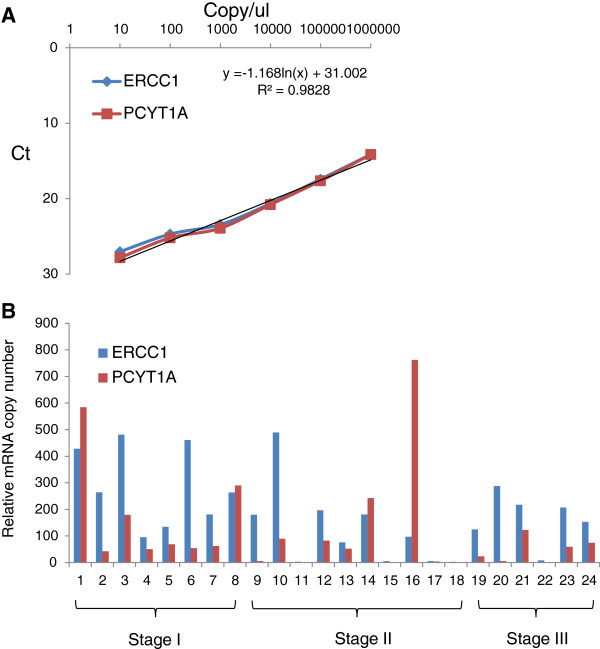
**qPCR analysis of mRNA expression profile for ERCC1 and PCYT1A genes on 24 lung cancer patient tissues. A**. qPCR standard. The experimental data using OriGene’s ERCC1 and PCYT1A qPCR standards. A formula for copy number calculation is shown on the chart. **B**. qPCR lung cancer tissue expression analysis. qPCRdata on OriGene’s TissueScan lung cancer cDNA array panel (HLRT104). The data were grouped based on cancer patient stages.

Quantitative real-time PCR can provide direct measurements of mRNAs level for different genes in the same tumor tissue sample. To ensure PCR amplification occurs at the same efficiency and to obtain an absolute mRNA copy number in the tissue mRNA samples, OriGene’s gene-specific qPCR copy number standards were serial-diluted and used for CT value measurement. The final data are summarized and plotted in Figure 5A. Our data show that the gene-specific probes chosen for ERCC1 and PCYT1A exhibit similar amplification efficiencies with their target genes. A copy number calculation formula was also created based on the standard curves.

To examine the gene expression levels for ERCC1 and PCYT1A in different cancer patient tissue samples, we performed qPCR analysis on OriGene’s lung cancer qPCR TissueScan cDNA array panel, which contains cDNA from 24 lung cancer patient tissues. The final result is presented in Figure 5B. The majority of samples show that the mRNA expression level for ERCC1 is higher than that of PCYT1A. However, four lung cancer patient samples exhibited higher PCYT1A mRNA expression levels than ERCC1 mRNA expression. This clearly demonstrates that in a certain lung cancer patient population, a significant amount of 8F1 immunoreactive signal could come from the 8F1-PCYT1A interaction and argues against the use of the 8F1 clone for ERCC1 IHC diagnostic assays. It also highlights the need to generate a highly mono-specific monoclonal antibody for ERCC1 clinical applications.

### Using a high density protein microarray chip to develop the most specific monoclonal anti-ERCC1 antibody for diagnostic IHC testing

To obtain highly mono-specific antibodies for ERCC1 IHC application, we used full-length human recombinant ERCC1 protein as the immunogen. This protein was purified from HEK293T cells that were transiently transfected with an ERCC1 cDNA clone (see Additional file 5: Figure S4 for coomassie staining of purified ERCC1). To ensure that we would generate the best IHC mAbs against ERCC1, we performed more than 8 fusions and got 18 positively validated hybridoma clones. All 18 mAbs were evaluated by Western-blotting the ERCC1 overexpression lysate and whole cell lysates of endogenous protein from 9 different cell lines. Our initial data showed that all 18 mAbs worked well with the ERCC1 overexpression lysate and four of them detected endogenous ERCC1 protein by Western blot with the cell line lysates (Additional file 6: Figures S5 and Additional file 7: Figure S6 show Western blot data for clones 4F9 and 2E12).

**Figure 6:**
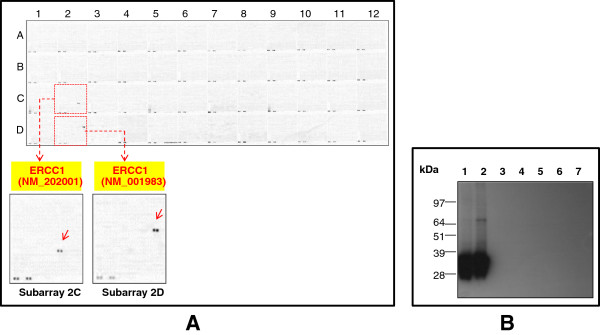
**To develop the most highly specific anti-ERCC1 monoclonal antibody with a protein microarray chip. A**. Specificity evaluation with a protein microarray chip. The overexpression protein microarray chip was immunostained with the 4F9 clone. This data shows that 4F9 is highly specific to ERCC1 (indicated with red arrows). No cross-reactivity was observed with any other test protein. **B**. Western Blot confirmation analysis. Seven OriGene VERIFY^TM^ overexpression lysate antigen standards (Lane 1 to 7) were fractionated on SDS-PAGE, and then immunoblotted with 4F9. The recombinant protein expression levels for the each of the different protein overexpression lysates were confirmed with the anti-DDK antibody (Figure 2B). Lanes 1 to 7 are loaded with samples for ERCC1 (NM_202001), ERCC1 (NM_001983), ERCC2 (NM_000400), ERCC3 (NM_000122), ERCC4 (NM_005236), ERCC5 (NM_000123) and PCYT1A (NM_005017).

**Figure 7:**
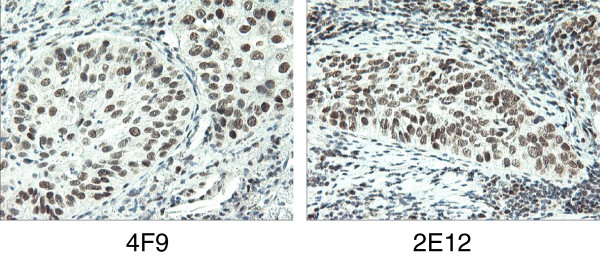
**The immunohistochemistry staining on NSCLC tissue sections with two newly developed mouse monoclonal anti-ERCC1 antibodies.** Pathologically-validated NSCLC FFPE tissue sections were used for IHC tests. All the IHC experiments were performed under the same antibody dilution factor (1:150) and incubation condition. The IHC data for two mouse monoclonal antibodies (Left: 4F9; Right: 2E12).

To identify the most specific anti-ERCC1 monoclonal antibodies for IHC application, we picked six different mAbs for protein microarray hybridization experiments. Although all of them can detect ERCC1 on the protein microarray chip, only three of them exhibited specificity without any cross-reactivity (Figure 6A and Additional file 8: Figure S7). Figure 6A is the representative protein microarray hybridization data on one of the clones, 4F9. The data were further confirmed by WB analysis (Figure 6B). To evaluate their performance in the IHC immunostaining application, two of the most specific clones, 4F9 and 2E12, were used on NSCLC FFPE tissue sections. Figure 7 shows that these two clones exhibit very distinct nuclear staining. Further experimental evidence demonstrated that the immunoassay performance for 2E12 clone is actually even better than 4F9 clone and exhibits much stronger binding affinity towards ERCC1 protein in native cell lines (Figure S6). In summary, we have successfully developed two highly specific anti-ERCC1 monoclonal antibodies for IHC pathological application.

## Discussion

Immunohistochemistry (IHC) is a widely used antibody-based assay for pathologists to semi-quantitatively measure tumor biomarker expression. Due to the nature of IHC assay itself, the quality of IHC data depends heavily on the quality of the antibody. Therefore, to develop a validated IHC assay for clinical usage, the first step is to identify antibodies with the highest specificity on their authentic targets. Clinical studies have shown that the level of ERCC1 gene expression in cancer patients is correlated to drug resistance of cisplatin-based chemotherapy [33]. Currently, there is no clinically approved ERCC1 IHC diagnostic assay available, likely due to the lack of a high quality anti-ERCC1 monoclonal antibody. Studies from a number of labs have shown that the 8F1 clone gives the best performance on NSCLC tissue in IHC assays [18,34]. However, Bhagwat, et al. claimed that 8F1 is not mono-specific [19]. By using an ERCC1 gene-ablated fibroblast cell line, they found that 8F1 can still binds to a specious immunoreactive band exhibiting a similar SDS-PAGE migration pattern as ERCC1. Interestingly, immunofluorescence experiments showed that this spurious 8F1 binding protein also localized in the nucleus. In this study, we have successfully identified PCYT1A, a nuclear membrane protein, as the 8F1 cross-reactive protein. We also found that PCYT1A exhibits similar biochemical and immunohistochemical properties as the spurious target discovered by Bhagwat et al. For example, PCYT1A shows a similar migration pattern as ERCC1 protein on SDS-PAGE gel even though it has a predicted molecular weight 10 kDa greater than ERCC1, and PCYT1A is localized exclusively in the nucleus by IHC. Alignment of the primary protein sequences showed no significant sequence homology between ERCC1 and PCYT1A, thus, it is likely that 8F1 recognizes a common conformational epitope on these two proteins. Although the 8F1 antibody binds equally well with ERCC1 or PCYT1A protein, there is no evidence that these two proteins share any functional relationship in development of drug resistance of cisplatin-based chemotherapy. Obviously, if a pathologist chose to use 8F1 antibody for ERCC1 protein expression evaluation, a potentially confounded result would be generated.

It is without question that high specificity is the pre-requisite for any antibody to be used for clinical diagnosis. Our data with 8F1 demonstrated that even a monoclonal antibody might not be mono-specific. Therefore, it is very critical to evaluate the specificity for every antibody to be utilized clinically. Scientists have spent years working to develop technologies to evaluate antibody specificity but we still do not have a platform that can address this question universally. At this stage, Western blot is the most commonly used approach to evaluate antibody specificity [35]. A single WB band at the right molecular weight is considered the primary criteria for antibody specificity. However, there are still a number of intrinsic issues that could lead to the wrong conclusion regarding antibody specificity. For example, the SDS-PAGE gel migration patterns for certain proteins, especially membrane proteins, do not always align well with their theoretically predicted molecular weights [36]. In another case, if a cross-reactive protein co-migrates with the authentic target on SDS-PAGE gel, it will be extremely difficult for us to distinguish them. For some target genes, the expression may be regulated in a highly temporal and spatial manner making an assessment by W. blotting uninformative from a clinical perspective [37,38]. Therefore, it is difficult to find a single type of cell line or tissue to evaluate antibody specificity in an unbiased way. The ultimate goal for antibody specificity testing is to evaluate whether a particular antibody will only bind to its authentic target, but not with other proteins within the human genome. Therefore, under the dream scenario, this antibody should be tested against every single human protein in the genome, which could be impossible to carry out practically. The protein microarray chip with its high throughput capacity provides a perfect technology platform. Since each protein target is spotted and annotated at a specific site on the chip, tens of thousands of target specific immuno-reactions can be performed simultaneously. In this study, we utilized OriGene VERIFY™ overexpression lysates collection for protein microarray chip generation. One of the major concerns is whether the endogenous protein expression within HEK293T cells will potentially create a background signal that masks the specific target on each crude lysate spot. According to our data, we found that the protein expression level for most of the exogenously expressed recombinant proteins is hundreds or even thousands times higher than their endogenous counterparts (data not shown). This is partly due to the utilization of a strong CMV promoter to boost the transcription level and to the presence of large T antigen within HEK293T that permits amplification of the transfected plasmids containing an SV40 replication origin. We also have data showing that a large number of membrane proteins, which are nearly impossible to purify, can be produced in large quantities in HEK293T cells and extracted efficiently under our lysis buffer conditions (data not shown). In this study, our experimental data clearly demonstrate that this chip is a perfect tool for antibody specificity testing. We believe that every potential diagnostic or therapeutic antibody should be evaluated on a genome-wide coverage protein microarray chip for mono-specificity before application in a clinical setting.

## Conclusion

In conclusion, we developed a high density protein microarray chip for antibody specificity evaluation. With this technology, we screened a number of commonly used anti-ERCC1 antibodies and discovered that 8F1, the most cited monoclonal antibody in clinical studies, is not specific. This antibody not only reacts with its authentic target, but also cross-reacts with PCYT1A, a nuclear membrane protein with no clinical implication on Cisplatin drug resistance. In the meantime, we also generated two IHC mouse monoclonal antibodies with exclusive target specificity, which were proofed by the high density protein microarray chip platform. Antibodies from these two clones, 2E12 and 4F9, also demonstrated superior performance on IHC application with NSCLC patient FFPE tissue sections.

## Competing interests

The authors declare competing financial interests. This research is partially supported by OriGene Technologies Inc. All the authors are currently full time employees from OriGene Technologies Inc.

## Authors’ contributions

DM and WH contributed to the designing of the experiments, data analyses and writing of the manuscript. DB printed the chip and performed protein microarray chip hybridization experiments. YS and TH performed the qPCR analysis. ZS contributed on TrueORF cDNA clone collection. DM and LM contributed on the lysate production. KM and ZL contributed on the IHC test. KY contributed on the anti-ERCC1 hybridoma generation. FW contributed on protein purification. WF contributed on 8F1 antibody serial dilution test. LM edited the paper. All authors read and approved the final manuscript.

## Supplementary Material

Additional file 1 **Figure S1.** The identification of polyclonal FL297 cross-reactive proteins with protein microarray chip. The high density protein microarray chip was immunostained with rabbit polyclonal anti-ERCC1 antibody FL297. The positive reactive proteins are pointed with red arrows. This data shows FL297 reacts strongly not only with its specific target (two ERCC1 transcript variants), but also two unrelated cytosolic proteins (FERMT3 and PDPK1). Click here for file

Additional file 2 **Figure S2.** Western blot analysis of FL297 with different overexpression lysates. The reactive lysates identified on Figure S1 were further analyzed by using rabbit polyclonal antibody FL297. Each lane was labeled accordingly. Click here for file

Additional file 3 **Figure S3A.** The immunohistochemistry staining on different normal human tissue sections with rabbit monoclonal anti-PCYT1A antibody. TMAs with 12 different tissue sections were immunostained by using rabbit monoclonal anti-PCYT1A antibody at 1:150 dilution. The representative IHC images for tissues with positive staining are shown here. Click here for file

Additional file 4 **Figure S3B.** The immunohistochemistry staining on different human carcinoma tissue sections with rabbit monoclonal anti-PCYT1A antibody. TMAs with 12 different carcinoma tissue sections were immunostained by using rabbit monoclonal anti-PCYT1A antibody at 1:150 dilution. The representative IHC images for tissues with positive staining are shown here. Click here for file

Additional file 5 **Figure S4.** Highly purified full length human recombinant ERCC1 protein used as immunogen. HEK293T cells were transiently transfected by using OriGene ERCC1 TrueORF gold cDNA clone. After transfection, the cells were culture at 37C for another 48 hrs before collection and lysis. The overexpressed recombinant ERCC1 protein was further purified by using anti-DDK affinity column. 0.5ug of purified ERCC1 was loaded for SDS-PAGE analysis. Click here for file

Additional file 6 **Figure S5.** Immunoblot analysis with 4F9 anti-ERCC1 monoclonal antibody. A. ERCC1 VERIFY^TM^ overexpression HEK293T cell lysate (Right lane) and empty vector negative HEK293T cell lysates (Left lane) were fractionated on SDS-PAGE and then immuoblotted with 4F9. B. Cell lysates prepared from 9 different cell lines were fractionated on SDS-PAGE gel and then immunoblotted with 4F9. Click here for file

Additional file 7 **Figure S6.** Immunoblot analysis with 2E12 anti-ERCC1 monoclonal antibody. A. ERCC1 VERIFY^TM^ overexpression HEK293T cell lysate (Right lane) and empty vector negative HEK293T cell lysates (Left lane) were fractionated on SDS-PAGE and then immuoblotted with 2E12. B. Cell lysates prepared from 9 different cell lines were fractionated on SDS-PAGE gel and then immunoblotted with 2E12. Click here for file

Additional file 8 **Figure S7.** Protein microarray chip hybridization with two newly developed mouse monoclonal anti-ERCC1 antibodies. The positive reactive proteins are pointed with red arrows. These data show that both clones are highly specific. A. Protein microarray chip hybridization data for clone 3 F6. B. Protein microarray chip hybridization data for clone 2E12. Click here for file
