# Supplementary figures and images for: Using protein microarray technology to screen anti-ERCC1 monoclonal antibodies for specificity and applications in pathology
Source: BMC Biotechnol. 2012 Nov 21;12:88. doi: 10.1186/1472-6750-12-88 (PMC3526464; doi:10.1186/1472-6750-12-88)

Donghui Ma, Figure S1

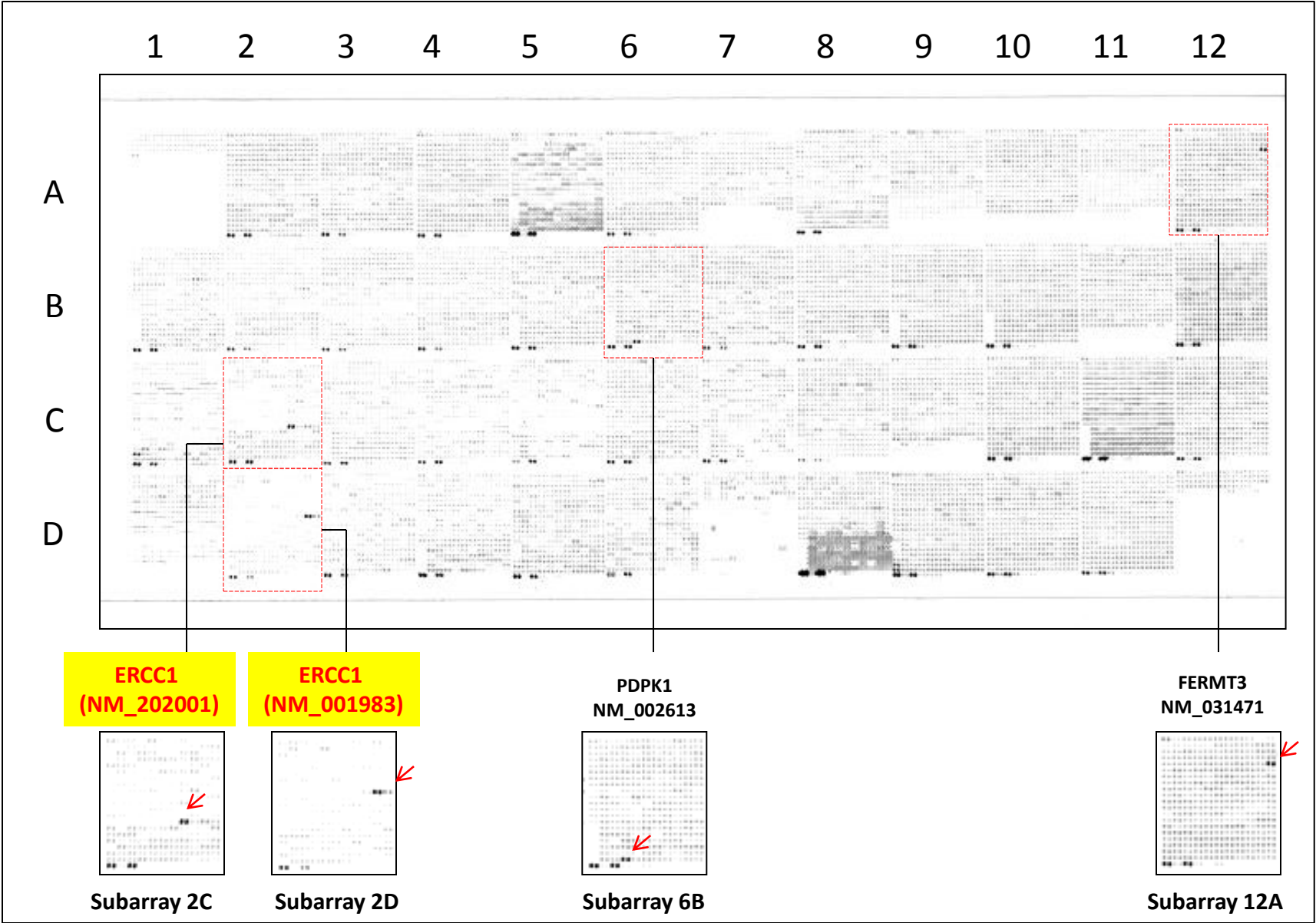

Supplement: Additional file 1 — Figure S1. The identification of polyclonal FL297 cross-reactive proteins with protein microarray chip. The high density protein microarray chip was immunostained with rabbit polyclonal anti-ERCC1 antibody FL297. The positive reactive proteins are pointed with red arrows. This data shows FL297 reacts strongly not only with its specific target (two ERCC1 transcript variants), but also two unrelated cytosolic proteins (FERMT3 and PDPK1). [file 1472-6750-12-88-S1.pdf]

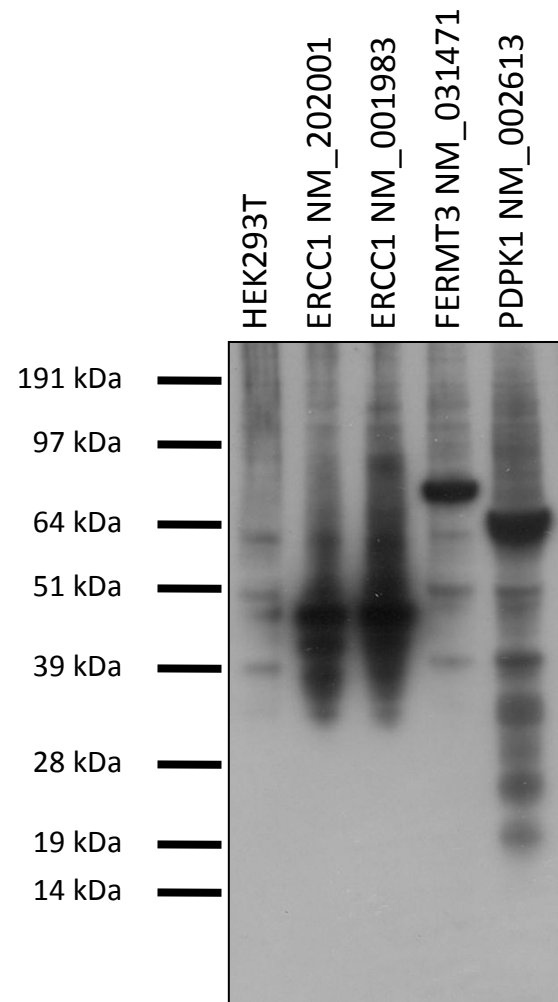

Supplement: Additional file 2 — Figure S2. Western blot analysis of FL297 with different overexpression lysates. The reactive lysates identified on Figure S1 were further analyzed by using rabbit polyclonal antibody FL297. Each lane was labeled accordingly. [file 1472-6750-12-88-S2.pdf]

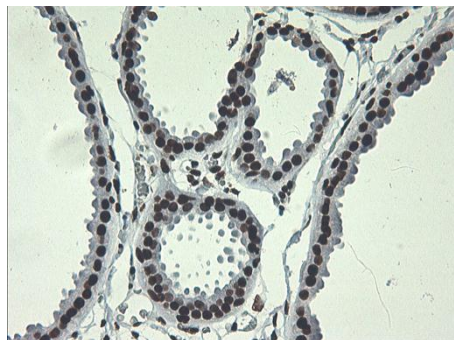

Breast

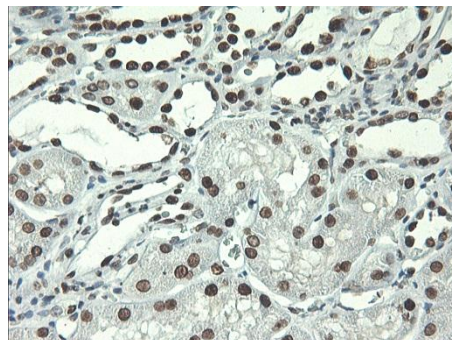

Kidney

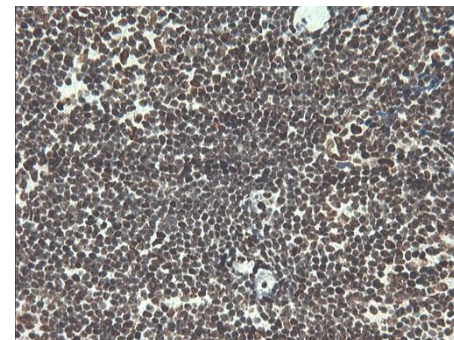

lymphoma

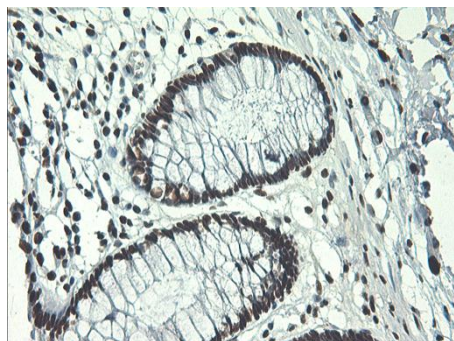

Colon

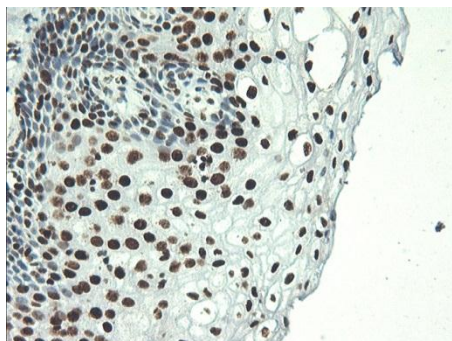

Tonsil

Supplement: Additional file 3 — Figure S3A. The immunohistochemistry staining on different normal human tissue sections with rabbit monoclonal anti-PCYT1A antibody. TMAs with 12 different tissue sections were immunostained by using rabbit monoclonal anti-PCYT1A antibody at 1:150 dilution. The representative IHC images for tissues with positive staining are shown here. [file 1472-6750-12-88-S3.pdf]

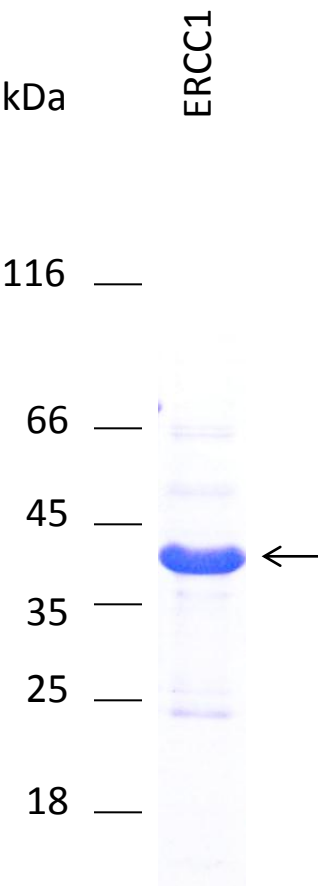

Supplement: Additional file 5 — Figure S4. Highly purified full length human recombinant ERCC1 protein used as immunogen. HEK293T cells were transiently transfected by using OriGene ERCC1 TrueORF gold cDNA clone. After transfection, the cells were culture at 37C for another 48 hrs before collection and lysis. The overexpressed recombinant ERCC1 protein was further purified by using anti-DDK affinity column. 0.5ug of purified ERCC1 was loaded for SDS-PAGE analysis. [file 1472-6750-12-88-S5.pdf]

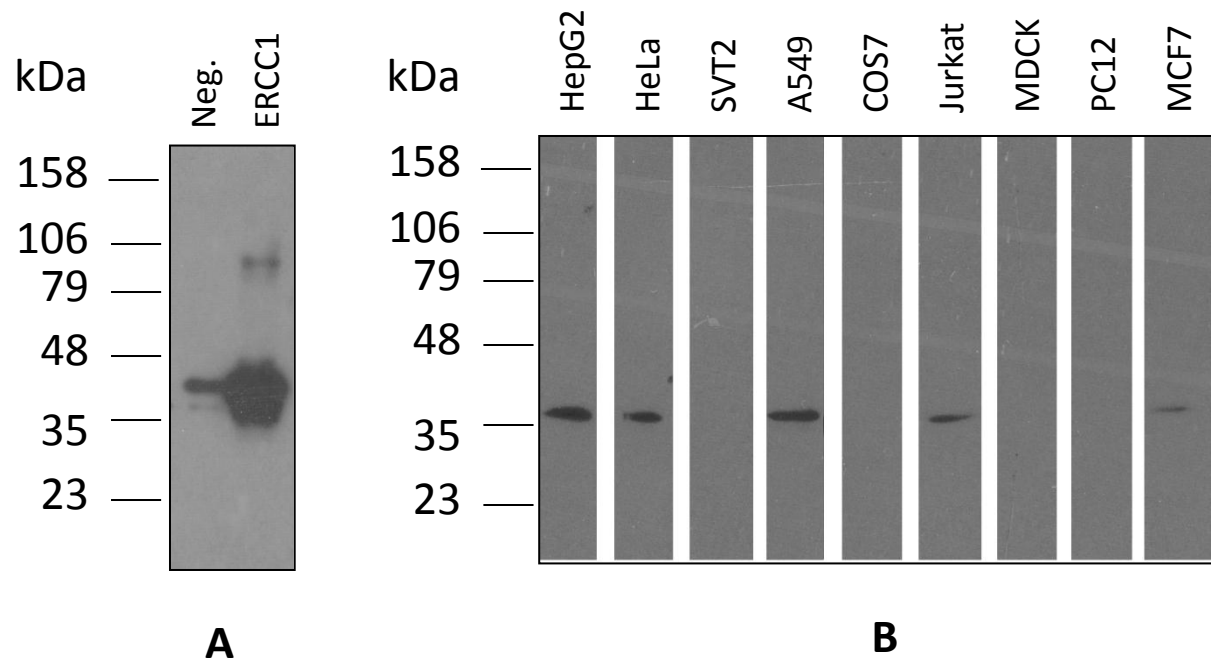

Supplement: Additional file 6 — Figure S5. Immunoblot analysis with 4F9 anti-ERCC1 monoclonal antibody. A. ERCC1 VERIFYTM overexpression HEK293T cell lysate (Right lane) and empty vector negative HEK293T cell lysates (Left lane) were fractionated on SDS-PAGE and then immuoblotted with 4F9. B. Cell lysates prepared from 9 different cell lines were fractionated on SDS-PAGE gel and then immunoblotted with 4F9. [file 1472-6750-12-88-S6.pdf]

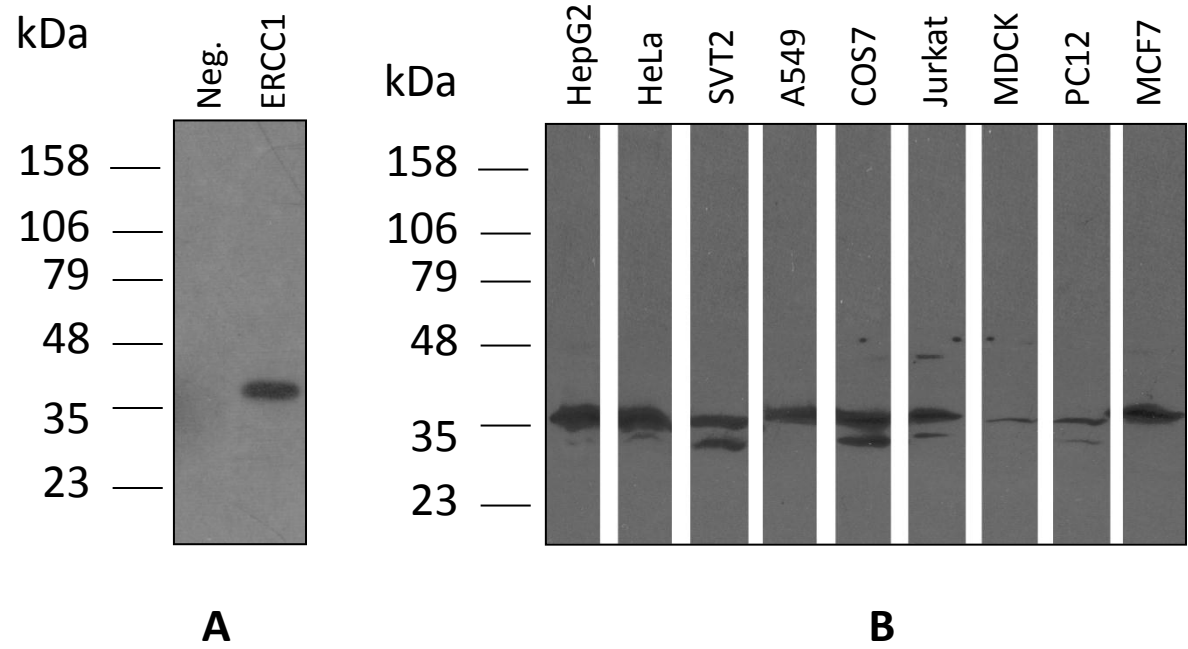

Supplement: Additional file 7 — Figure S6. Immunoblot analysis with 2E12 anti-ERCC1 monoclonal antibody. A. ERCC1 VERIFYTM overexpression HEK293T cell lysate (Right lane) and empty vector negative HEK293T cell lysates (Left lane) were fractionated on SDS-PAGE and then immuoblotted with 2E12. B. Cell lysates prepared from 9 different cell lines were fractionated on SDS-PAGE gel and then immunoblotted with 2E12. [file 1472-6750-12-88-S7.pdf]

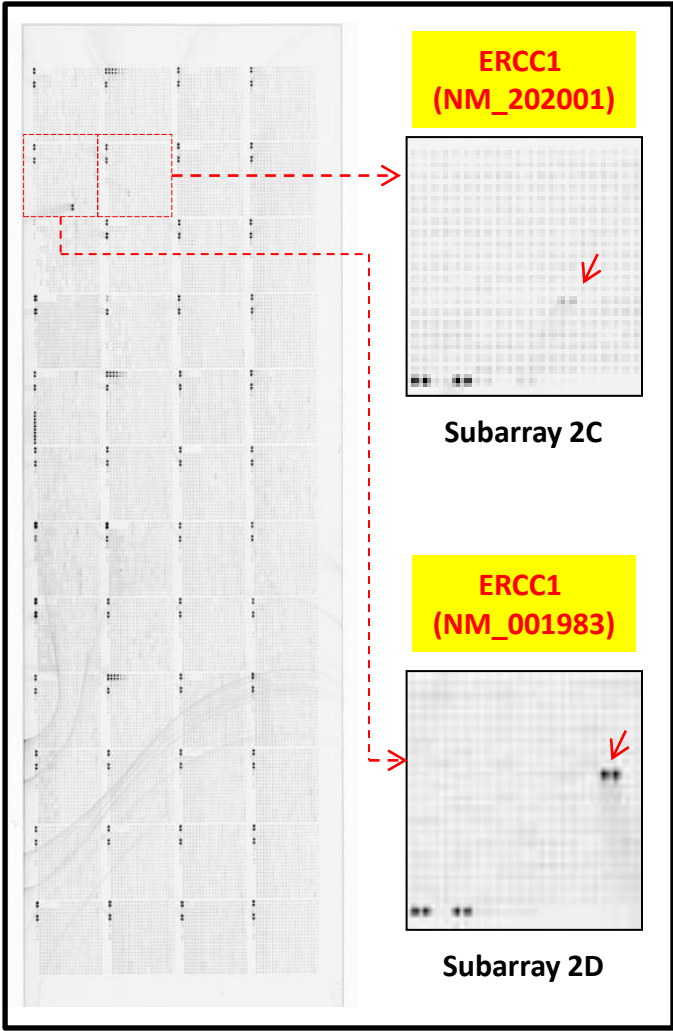

A

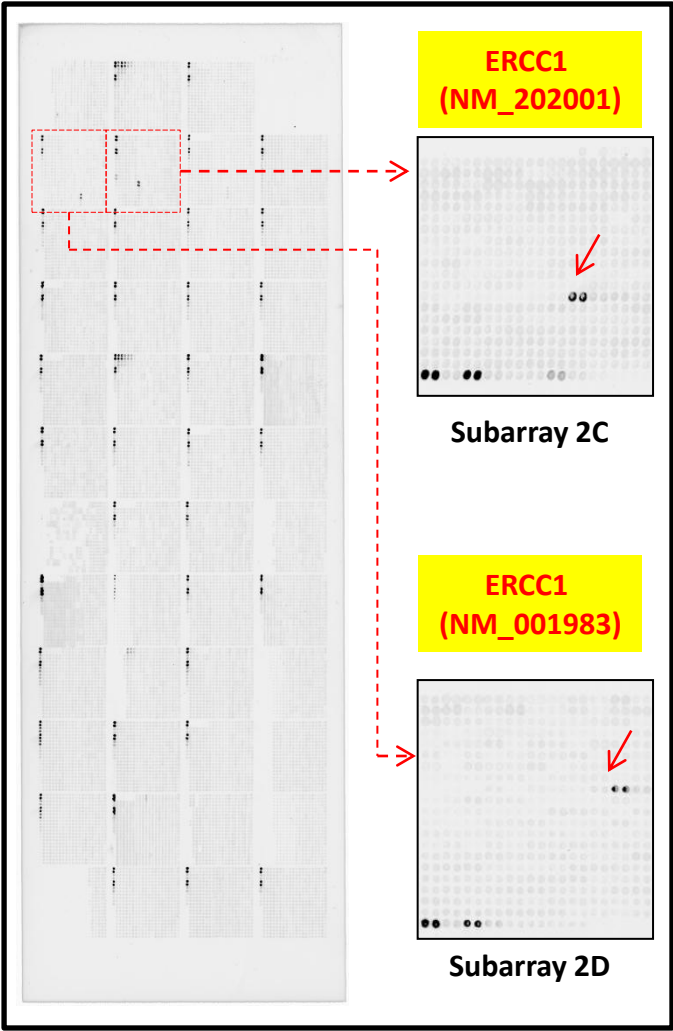

B

Supplement: Additional file 8 — Figure S7. Protein microarray chip hybridization with two newly developed mouse monoclonal anti-ERCC1 antibodies. The positive reactive proteins are pointed with red arrows. These data show that both clones are highly specific. A. Protein microarray chip hybridization data for clone 3 F6. B. Protein microarray chip hybridization data for clone 2E12. [file 1472-6750-12-88-S8.pdf]
