# Supplementary material for: Using protein microarray technology to screen anti-ERCC1 monoclonal antibodies for specificity and applications in pathology
Source: BMC Biotechnol. 2012 Nov 21;12:88. doi: 10.1186/1472-6750-12-88 (PMC3526464; doi:10.1186/1472-6750-12-88)
Supplement: Additional file 4 — Figure S3B. The immunohistochemistry staining on different human carcinoma tissue sections with rabbit monoclonal anti-PCYT1A antibody. TMAs with 12 different carcinoma tissue sections were immunostained by using rabbit monoclonal anti-PCYT1A antibody at 1:150 dilution. The representative IHC images for tissues with positive staining are shown here. [file 1472-6750-12-88-S4.pdf]

Donghui Ma, Figure S3B

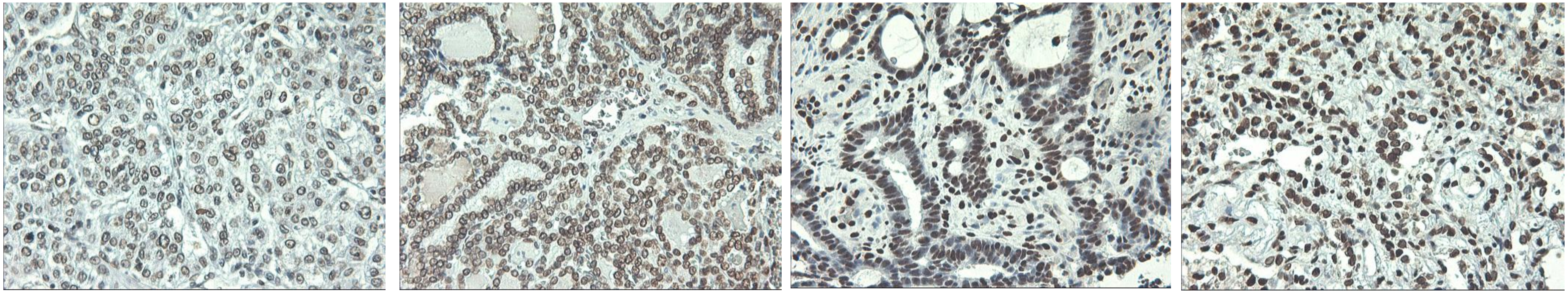

Carcinoma of liver | Carcinoma of thyroid | Adenocarcinoma of colon | Carcinoma of lung

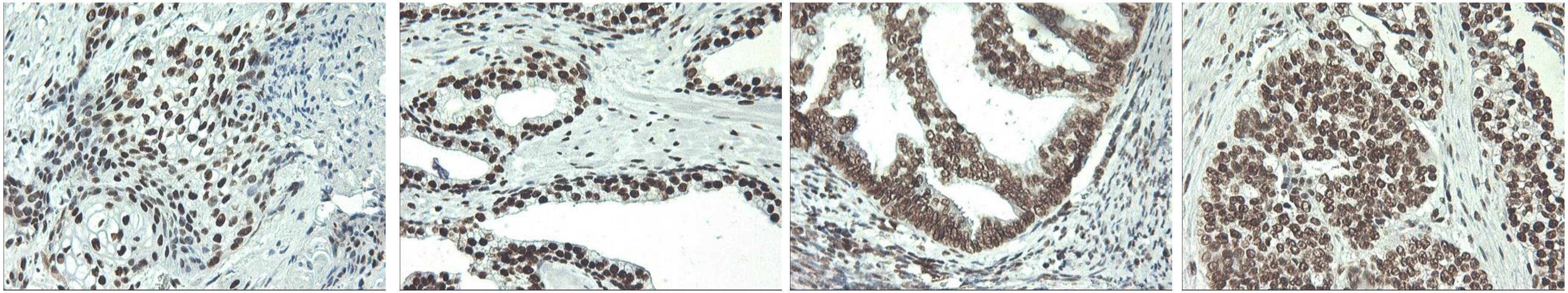

Carcinoma of bladder | Carcinoma of Prostate | Adenocarcinoma of endometrium | Adenocarcinoma of ovary
